# Assessment of a Newly Developed, Active Pneumatic-Driven, Sensorimotor Test and Training Device

**DOI:** 10.3390/s141224174

**Published:** 2014-12-15

**Authors:** Wolfram Haslinger, Lisa Müller, Esmeralda Mildner, Stefan Löfler, Helmut Kern, Christian Raschner

**Affiliations:** 1 Department of Sport Science, University of Innsbruck, Fuerstenweg 185, A-6020 Innsbruck, Austria; E-Mails: wolfram.haslinger@gmx.at (W.H.); lisa.mueller@uibk.ac.at (L.M.); m_essi@gmx.at (E.M.); 2 Ludwig Boltzmann Institute of Electrical Stimulation and Physical Rehabilitation, Wilhelminenspital, Montleartstraβe 37, A-1160 Vienna, Austria; E-Mails: stefan.loefler@wienkav.at (S.L.); wil.pys.kern-forschung@wienkav.at (H.K.); 3 Institute for Physical Medicine and Rehabilitation, Wilhelminenspital, Montleartstraβe 37, A-1160 Vienna, Austria

**Keywords:** postural balance, exercise therapy, reproducibility of results, feedback training

## Abstract

The sensorimotor system (SMS) plays an important role in sports and in every day movement. Several tools for assessment and training have been designed. Many of them are directed to specific populations, and have major shortcomings due to the training effect or safety. The aim of the present study was to design and assess a dynamic sensorimotor test and training device that can be adjusted for all levels of performance. The novel pneumatic-driven mechatronic device can guide the trainee, allow independent movements or disrupt the individual with unpredicted perturbations while standing on a platform. The test-reliability was evaluated using intraclass correlation coefficient (ICC). Subjects were required to balance their center of pressure (COP) in a target circle (TITC). The time in TITC and the COP error (COPe) were recorded for analysis. The results of 22 males and 14 females (23.7 ± 2.6 years) showed good to excellent test-retest reliability. The newly designed Active Balance System (ABS) was then compared with the Biodex Balance System SD^®^ (BBS). The results of 15 females, 14 males (23.4 ± 1.6 years) showed modest correlation in static and acceptable correlation in dynamic conditions, suggesting that ABS could be a reliable and comparable tool for dynamic balance assessments.

## 1. Introduction

The sensorimotor system (SMS) plays an important role in sports and in our ability to manage every day activities. Injuries [1], degenerative diseases [2], ageing [3], and low physical activity [4] affect the SMS and result in a disturbed function. An impaired SMS has been determined as the leading cause of falls [5,6]. Exercise programs designed to improve balance appear to be promising in rehabilitation and the prevention of injuries [7,8].

In most clinical and therapeutic settings, testing procedures measure postural control upright in either static or dynamic conditions [9]. Baloh and coworkers [10] showed that static balance assessments do not detect balance disorder as clearly as dynamic tests. Turbanski and Schmidtbleicher [11] also suggested that posturography in static conditions cannot predict dynamic performance.

Various dynamic balance assessment tools have been designed. Many of them are passive by nature or have major shortcomings in regard to individualization, variability and continuous gain during the training process. Some are complicated to use and sometimes safety is an issue. Only view balance assessment tools generate active perturbations [12].

The challenges of developing a dynamic balance test and training device is the difficulty of measurement, and the reproducibility of the results. Test-retest reliability of balance tests have shown mixed results from poor [13] to acceptable correlations [8].

The aim of the present study was to assess an adaptable and upgradable, active, dynamic test and training device that can be adjusted for all levels of performance. For this purpose, a pneumatic driven mechatronic platform (on which the subject stands) was designed to allow either free, guided or disrupted movement of the training individual during the exercises. Second, the test–reliability of the portable easy-to-use Active Balance System (ABS) was evaluated (Study 1). Third, the ABS was compared to the Biodex Balance System SD^®^ (BBS, Biodex, Inc., Shirley, NY, USA), which is another well-investigated dynamic balance tool [14–16] (Study 2).

## 2. Methods

### 2.1. Study 1

#### 2.1.1. Test device (ABS)

**Figure 1.**
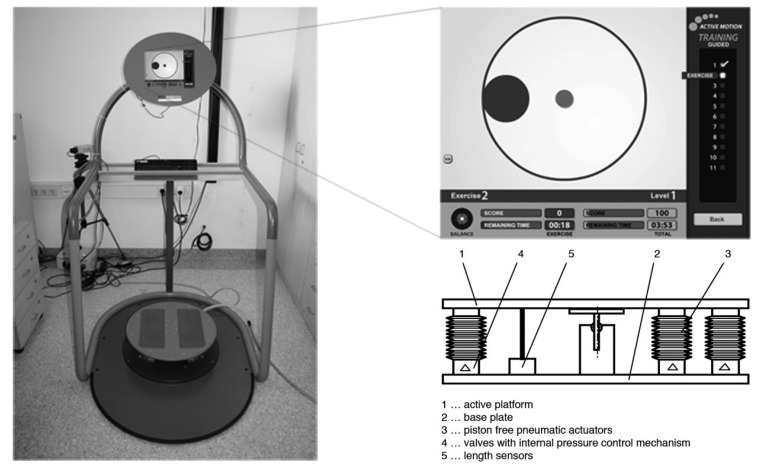
Test and training device.

The device (Figure 1) consists of a steel frame, which serves as a security rail for the test person, and functions as a support element for a PC unit, with an integrated 15 in. touch screen. An external control box regulates the power supply and data communication between the action unit and the PC. Standard compressors supply the training device with air (6 bars). The active platform designed by FerRobotics (FerRobotics Compliant Robot Technology GmbH, Linz, Austria) has a diameter of 530 mm. The platform is provided with skid-resistant pads and positional marker. The three piston-free pneumatic actuators (PFPA), are evenly distributed on the circle, set the platform in motion. The PFPA are countervailed by three springs, which are centrally located between the actuators. The PFPA allow for active as well as passive combinations of movements. The movements can be controlled by continuously adjusting the amplitudes and frequencies.

Two axles allow the platform to tilt ±12° in two planes. The PFPA function both as actuators and force sensors. The actuation is directed by valves and an internal pressure control mechanism. The forces acting on the three PFPA are estimated from the pressure and length of the PFPA, following a nonlinear characteristic diagram, coming from the producer. Three length sensors are implemented for that calculation, allowing at the same time to determine the exact location of the plate. A control algorithm calculates the restoring force and the force application point of the user's center of pressure (COP) in roll (x) and pitch (y) direction. This calculation is based upon the known forces acting on each PFPA and their perpendicular distance to the coordinate axes. The COP is then calculated by equilibrium equations, as the resulting force application point to reach static balance. The data are sampled at 1000 Hz. The test results are then saved in the PC unit, and the touch screen displays a visual user feedback. All data are available for analyses. User can store their test results in a high score section. A more detailed description of the hardware is given in patent number AT 502520, which has been registered at the Austrian patent office [17].

All software components are designed by Imagination (Imagination Computer Services GesmbH, Vienna, Austria). The user interface is divided in four menu items: “Warm-up”, “Test”, “Training”, and “Games”. Individuals are asked to maneuver a COP dot (the small gray dot which represents the body's COP) into a target circle (black circle, 2.5 times bigger than the COP dot) by shifting their body mass. The individuals can choose from 11 different predefined exercises (Table 1), three different modes, and two different performance levels. The “supported” mode allows for the platform to follow all the movements thus assists the individual with the performance of the movement tasks. The platform actively maneuvers the individual's COP dot into the system's displayed target circle (Video 1). The elasticity of the springs determines the movement of the platform when using the “independent” mode setting. When choosing the “advanced” mode, the platform hinders the individual and interferes with the control movements. The platform actively maneuvers the individual's COP dot away from the system's displayed target circle (Video 2).

The system can be programmed and adjusted continuously and the movement tasks can be overlaid with five pre-programmed intervening movement patterns.

The individuals can choose between two levels of difficulty. When choosing level 1, the target circle is 2.5 times bigger than the COP dot. In level 2, t the target circle is 2 times bigger than the COP dot. The COP parameters and the time needed for the COP dot to overlap 75% of the target circle can be calculated.

#### 2.1.2. Participants

A total of 36 Sport Science students (14 females, 22 males), with a mean age of 23.7 ± 2.6 years, a mean height of 176.2 ± 8.0 cm and a mean body mass of 69.2 ± 9.4 kg took part in the test-retest reliability study. All subjects were asked to refrain from any type of athletic activities during the duration of the study. The participants were tested on the training device after an introduction to the subject matter and were informed about any potential risks. Only healthy test subjects were recruited.

#### 2.1.3. Testing Procedure

A five min warm-up phase on the bike ergometer followed by a balance-oriented warm-up program preceded each one of the testing phases. To minimize learning effects, the warm-up program occurred on testing platforms that were similar to the ABS. The participants were asked to choose a standardized starting position, *i.e.*, two-legged hip-wide stance, with slightly flexed ankles and knees. Their arms hung loosely at their sides and could be used to maintain balance. All tests occurred under laboratory conditions and were conducted without shoes.

**Figure 2.**
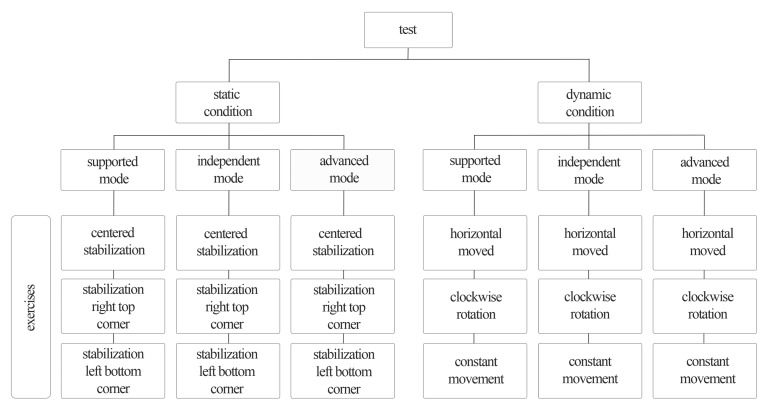
Schema of the testing process on the ABS.

Three different exercises were performed under two test conditions in three different modes: “supported”, “independent”, and “advanced”. The static conditions were as follows: (i) centered stabilization; (ii) stabilization right top corner; and (iii) stabilization left bottom corner. The dynamic conditions were as follows: (i) horizontally moved; (ii) clockwise rotation; and (iii) constant movement (Figure 2). Each exercise lasted 30 s with a 20 s break in between the exercises. The participants took a 1 min break before changing to a different mode. Target size was set at 2.5 times the COP dot, and the target coverage was set at 75%. To assess the reliability of the outcome measurements, a test-retest design was used.

#### 2.1.4. Data Analysis

**Figure 3.**
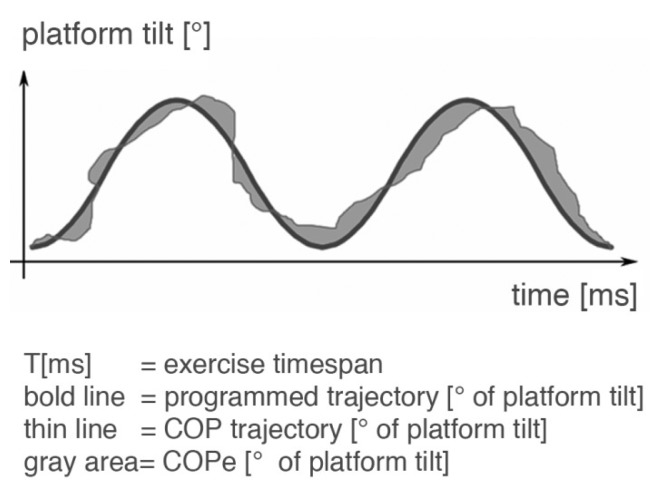
Center of pressure error (COPe). The COPe [°] is calculated from the deviation between the person's effective actual COP trajectory and the programmed trajectory in the given exercise time span. A maximum 24° of erroneous tilt of platform is possible.

For ABS, the time in which the COP dot was inside the target circle (TITC) relative to the test period was used to analyze balance control. The center of pressure error (COPe) data was also calculated (Figure 3).

(1) Error e[°]=COP trajectory[°]−programmed trajectory[°]

(2) $$\text{COPe}\left[{}^{\circ}\right]=\frac{1}{T\left[ms\right]}\int_{0}^{t\left[ms\right]}\left|e\left[{}^{\circ}\right]\right|dt$$

To assess the reliability of the outcome measurements, the intraclass correlation coefficient (ICC) and the standard error of measurement (SEM) were calculated [18,19]. In the absence of a normal distribution, the correlation was calculated according to Spearman. Based on the design of the study, a model with mixed two-way effects was chosen for the ICC. The person effects are selected randomly while the measuring effects are set values (ICC 3.1) [19,20]. According to Weir [18], the SEM and the minimum detectable change (MDC) were calculated with the following formula: $\text{SEM}=\text{SD}\sqrt{1-\text{ICC}}$; MDC= SEM × 1.96.

For the comparison of absolute reliability between the COP measurements, the coefficient of variation (CV; mean CV from individual CVs) was determined as follows: CV = SD/mean × 100. The significance level was set at *p* < 0.05 for all tests. Point estimates of the ICC were interpreted as follows: (0.00–0.39) poor, (0.40–0.59) fair, (0.60–0.74) good and (0.75–1.00) excellent [21].

All normal distribution was tested via Kolmogorov-Smirnov-Test. The data were processed using the Statistical Package for the Social Sciences (SPSS Inc. Version 18.0, Chicago, IL, USA).

### 2.2. Study 2

#### 2.2.1. Test Device (BBS)

In the second study, ABS was compared to BBS. The BBS consisted of a multiaxial platform. The passively 360° moveable platform allowed up to 20° of surface tilt. The resistance of the platform was adjustable from spring resistance eight (the most stable setting) to one (the least stable setting). The system generated three indices: the medial-lateral stability index (MLSI), the anterior-posterior stability index (APSI), and the overall stability index (OSI) [14]. The BBS provided three standardized testing protocols, including the postural stability test, the limits of stability (LOS) test, and a fall risk test.

#### 2.2.2. Participants

For the comparison with the BBS, 29 students (15 females, 14 males), with a mean age of 23.4 ± 1.6 years, a mean height of 175.5 ± 9.0 cm and a mean body mass of 68.5 ± 11.5 kg took part in the study.

#### 2.2.3. Testing Procedure

All tests were performed under the same conditions and the same warm-up procedure and standardized starting position as in study one. To determine the static stability, a 30 s postural stability test was performed. The (LOS) was used to measure balance under dynamic conditions. Balance was assessed at the spring resistance level of 3. The performance requirements for ABS were adapted from the BBS testing procedure. Static balance was assessed using exercise 10 “centered stabilization” for 30 s. The target size was set at 2.0 times the COP dot, and the target coverage at 100%. For dynamic conditions, the exercise 8 “pop up random location” was chosen. The target size was set at 2.5, and the target coverage was set at 100%. Balance was measured for 100 s. The tests under the static conditions were performed first on both types of equipment. Upon completion of an additional familiarization trial the students were randomly assigned to their respective groups, which were either BBS or ABS. The testing order was randomized.

#### 2.2.4. Data Analysis

For the ABS the same parameters were calculated as in study one. The MLSI, the APSI, the OSI for the static test, and the time to finish the dynamic test were calculated for BBS [14]. The comparison of ABS to BBS was assessed using Pearson and Spearman (absence of normal distribution) correlation coefficient respectively. The normal distribution was tested *via* Kolmogorov-Smirnov-Test. The local Institutional Review Board of the Department of Sport Science of the University of Innsbruck approved these studies. All participants provided informed consent. Testing was carried out according to the Declaration of Helsinki.

## 3. Results

### 3.1. Study 1

The detailed results of the test-retest reliability of the three different exercises under the static and dynamic conditions are provided in Tables 2 and 3.

In the three different modes, ABS showed good to excellent test-retest reliability (ICC total = 0.60–0.85; total: summary of the three different exercises). Under supported conditions, the TITC and COPe values did not reach an ICC value of 0.6 (except ICC COPe stat = 0.61). The individual variation (CV) values of TITC were between 0.89% (total supported static conditions) and 30.02% (total advanced static conditions), suggesting that variation increases under more difficult conditions. By contrast, the CV values of COPe total were lowest in the advanced mode under the dynamic conditions (CV range = 13.73%–32.89%). The SEM values and MDC values were between (SEM total range = 0.79%–13.09%) and (MDC total range = ±0.46 s–±3.55 s) for TITC and (SEM total range = 8.3%–19.43%) and (MDC total range = ±0.24°–±0.8°) for the COPe in the static and dynamic conditions.

### 3.2. Study 2

Table 4 presents the results of the comparison of the two balance systems. The results showed a modest correlation between COPe measures of ABS and the balance measures of BBS in static conditions. In dynamic conditions, the comparison of LOS and the customized test on ABS showed an acceptable result for the TITC.

## 4. Discussion

Balance and postural control is a fundamental basis for human stance, and movement. Adequate postural control depends on sensory information from proprioceptive, vestibular, cutaneous and visual sources. The central nervous system plans and executes appropriate muscular activation patterns based on this information [22]. Regardless of age or performance level, balance should be trained and tested.

The aim of the study was to assess the test-retest reliability of a newly developed active test and training device in static and dynamic conditions. In a second step, the system was compared with another widely used dynamic balance tool. The results showed that the ABS exhibits good to excellent test-retest reliability for the TITC and COPe. The ABS showed concurrent validity with the BBS.

Most of the previous studies evaluated test-retest reliability and validity by focusing on balance test protocols without any active help or disturbance. These studies showed acceptable reliability and validity in “inactive” testing and training settings [13,23,24]. The present study exhibited comparable results in the independent mode (ICC_total_ = 0.60–0.85).

In the supported mode, the ICC _TITC_ values were poor (ICC_total TITC_ = 0.10–0.21) in both the static and dynamic conditions. Because the ICC values are prone to inter-individual variation, e.g., large variation between subjects effects high retest correlation and vice versa, within-individual variation was calculated using SEM and CV [25]. In contrast to the results of ICC, SEM and CV showed acceptable results (SEM_total TITC_ = 0.79%–3.81%, CV_total TITC_ = 0.89%–4.00%). This finding may result from a low demand on the balance ability of healthy sport science students in the supported conditions. In addition, the total MDC titc in both conditions in the three different modes (MDC_total TITC_ = ±0.46 s–±3.55 s) suggested an acceptable level of the measurement error of ABS scores. MDC represents the boundaries of the clinically significant change that can be expected after a treatment [18]. The ICC_total TITC_ in the advanced mode in the static conditions is interpreted with caution because of the high CV value (CV_total TITC_ = 30.02%), suggesting a poor “absolute” reliability. A possible explanation for this could be that the perturbations were applied in the most challenging mode and therefore had more impact on the inter-individual balance ability. According to Domholdt [26], reliability is dependent on the studied population, therefore further research should examine the reliability of the ABS results among sedentary people in the supported mode (age, history of injury) and athletes from various sports in the “advanced” mode.

The comparison of measures from dynamic balance systems is not easy to assess. This may be due to problems in determining which criterion measure of dynamic balance should be compared [15]. Gstöttner *et al.* [16] investigated the balance ability of the preferred and non-preferred leg of soccer players using BBS, the Tetrax Interactive Balance System^®^ (Neurodata, Wien, Austria) and the Equitest System^®^ (NeuroCom, Clackamas, OR, USA), showing mixed results. This may be due to the complexity of the balance demands so comparing dynamic balance abilities can be criticized. The problem of comparing balance measurements must be taken in account when interpreting our data.

COPe was chosen because the calculations are analogous to the BBS. Both are based on degrees of tilt over anterior-posterior and medial-lateral axes. The results are similar to the TITC values. COPe represents an even more precise measurement and is comparable to the sway area, which is a reliable COP measurement [27]. The results represented in the BBS postural stability test showed modest correlations with the ABS values in the static conditions (r_TITC_ (27) = −0.52, *p* < 0.01; r_COPe_(27) = 0.60–0.62, *p* < 0.001). Conversely, in the dynamic conditions, the comparison of LOS with the customized test of ABS showed an acceptable result (r_TITC_ (27) = −0.72, *p* < 0.001). In addition, ABS indicated comparable ICC values for total COPe values in the advanced mode (ICC_total COPe_ = 0.64–0.84), in relation to the similar OSI values (BBS) as reported by Cachupe *et al.* [15]. In the present study OSI was used because previous studies showed that OSI is the most reliable index [14–16].

## 5. Conclusions

In conclusion, ABS could be a reliable dynamic test and training device to detect differences or changes in balance in healthy young participants. The results of the present study showed good to excellent test-retest reliability. The system provides comparable data to BBS when assessing COPe and the TITC.

ABS can offer a safe, effective and varied training for all levels of performance. The system is able to detect the personal training threshold, so individuals can be optimally guided through the different training levels. The training can be designed to challenge and motivate.

The ABS offers a wide spectrum of COP measurements. Several studies have shown that among COP parameters, mean total velocity is the most reliable value, suggesting that this parameter is a good predictor for quantifying balance performance [27,28]. Further research should be performed to examine the reliability of various types of measurement and among groups of varying ages, health or activity levels.

## Figures and Tables

**Table 1.** Exercises of the Active Balance system.

**Name of Exercise**	**Description of Exercise**
1 POP UP TOP/BOTTON	The COP dot has to be moved as quickly as possible into the target circle, which appears alternately above and below.
2 POP UP LEFT/RIGHT	The COP dot has to be moved as quickly as possible into the target circle, which appears alternately left and right.
3 VERTICALLY MOVED	The COP dot has to follow the movement and vertically change the position of the target circle as accurately as possible.
4 HORIZONTALLY MOVED	The COP dot has to follow the movement and horizontally change the position of the target circle as accurately as possible.
5 CLOCKWISE ROTATION	The target circle is moving clockwise, and the COP dot has to follow its changing position as accurately as possible.
6 COUNTERCLOCKWISE ROTATION	The target circle is moving counterclockwise, and the COP dot has to follow its changing position as accurately as possible.
7 CONSTANT MOVEMENT	The target circle is moving diagonally without acceleration from the top left corner to the bottom right corner and back. The COP dot has to follow its changing position as accurately as possible.
8 POP UP RANDOM LOCATION	The COP dot has to be moved as quickly as possible into the target circle, which appears randomly in the operating space.
9 ACCELERATED MOVEMENT	The target circle is moving freely and with accelerated speed. The COP dot has to follow its changing position as accurately as possible.
10 CENTERED STABILIZATION	The COP dot has to be stabilized in the center of a target circle.
11 OFF-CENTER STABILIZATON	The COP dot has to be stabilized in the center of an off-centered target circle.

**Table 2.** Test-retest reliability analysis of the TITC in three different exercises and three different conditions of postural difficulty (*n* = 36).

**TITC [s] Static Conditions**											

**Mode**	**Test *M***	***SD***	**Retest *M***	***SD***	**ICC**	**(CI)**	**SEM**	**SEM (%)**	**± MDC**	**CV (%)**	**(CI)**
support											
centered	29.86	0.40	29.88	0.20	0.36*	(*p* = 0.041)					
right top corner	29.10	0.37	29.02	0.70	0.24	(−0.107–0.539)	0.38	1.31	0.75	1.51	(1.21–2.00)
left bottom corner	29.04	0.42	29.06	0.45	0.26	(−0.098–0.551)	0.30	1.02	0.58	1.19	(0.95–1.57)

total	29.33	0.28	29.32	0.39	0.21	(−0.147–0.517)	0.23	0.79	0.46	0.89	(0.72–1.18)

independent											
centered	27.80	2.36	28.60	1.60	0.61	(0.320–0.794)	1.14	4.04	2.23	6.49	(5.19–8.56)
right top corner	26.51	1.67	27.10	3.33	0.41 *	(*p* = 0.019)					
left bottom corner	26.59	2.83	27.65	1.44	0.47**	(*p* = 0.006)					

total	26.97	1.99	27.78	1.86	0.85	(0.254–0.950)	0.74	2.69	1.45	6.89	(5.50–9.08)

advanced											
centered	11.71	4.59	13.45	4.41	0.75	(0.443–0.883)	2.14	16.98	4.19	33.96	(24.59–40.55)
right top corner	11.18	4.28	13.25	4.22	0.58	(0.243–0.781)	2.50	20.43	4.89	31.52	(23.13–38.14)
left bottom corner	12.43	3.72	13.37	4.14	0.71	(0.484-0.844)	1.98	15.32	3.87	28.30	(21.10–34.80)

total	11.77	3.88	13.36	3.92	0.81	(0.402–0.925)	1.64	13.09	3.22	30.02	(22.19–36.60)

**TITC [s] Dynamic Conditions**											

support											
horizontal moved	24.29	1.59	24.88	0.66	0.17	(−0.124–0.456)	0.85	3.47	1.67	3.82	(3.09–4.98)
clockw. rotation	26.52	2.98	27.69	1.76	0.08	(−0.211–0.381)	1.72	6.35	3.37	6.63	(5.35–8.63)
const. movement	25.77	2.18	27.43	1.09	−0.16	(−0.394–0.128)	1.15	4.33	2.26	4.02	(3.25–5.24)

total	25.53	1.80	26.67	0.80	0.10	(−0.147–0.361)	0.99	3.81	1.95	4.00	(3.24–5.22)

independent											
horizontal moved	16.20	3.73	18.42	3.20	0.59	(0.117–0.807)	2.06	11.89	4.03	18.50	(14.50–23.37)
clockw. rotation	15.50	4.68	17.68	2.97	0.44	(0.116–0.673)	2.54	15.34	4.99	20.46	(15.92–25.67)
const. movement	19.37	3.90	21.13	3.78	0.70	(0.340–0.855)	1.99	9.82	3.90	17.81	(13.99–22.55)

total	17.02	3.66	19.08	2.83	0.66	(0.102–0.858)	1.81	10.03	3.55	17.20	(13.47–21.71)

advanced											
horizontal moved	12.49	3.85	14.60	3.22	0.50	(0.140–0.730)	2.23	16.44	4.37	23.30	(17.93–28.90)
clockw. rotation	11.97	3.53	13.51	2.96	0.54	(0.228–0.744)	1.97	15.48	3,87	22.83	(17.60–28.37)
const. movement	10.49	2.31	12.02	2.32	0.55	(0.104–0.777)	1.42	12.64	2.79	18.76	(14.69–23.68)

total	11.65	2.43	13.37	2.08	0.60	(−0.20–0.841)	1.34	10.73	2.63	17.03	(13.41–21.62)

TITC: time in target circle; ICC: intraclass correlation coefficient; CI: 95% confidence interval; SEM: standard error of measurement; MDC: minimal detectable change; CV: coefficient of variation; clockw.: clockwise; const.: constant. total: summary of the three different exercises. Spearman's rank correlation coefficient in absence of normal distribution:

** significance level (*p* < 0.01);

* significance level (*p* < 0.05).

**Table 3.** Test-Retest reliability analysis of the COP error in three different exercises and three different conditions of postural difficulty (*n* = 36).

**COPe [°] Static Conditions**											

**Mode**	**Test *M***	***SD***	**Retest *M***	***SD***	**ICC**	**(CI)**	**SEM**	**SEM (%)**	**± MDC**	**CV (%)**	**(CI)**
support											
centered	0.27	0.11	0.23	0.08	0.50**	(*p* = 0.003)					
right top corner	0.30	0.13	0.30	0.14	0.48**	(*p* = 0.005)					
left bottom corner	0.28	0.10	0.27	0.08	0.13	(−0.226–0.454)	0.06	23.46	0.12	25.19	(19.06–31.42)

total	0.28	0.11	0.27	0.09	0.61**	(*p* = 0.000)					

independent											
centered	0.58	0.23	0.55	0.25	0.60	(0.327–0.779)	0.14	24.23	0.27	38.22	(27.00–44.53)
right top corner	0.73	0.27	0.65	0.33	0.62	(0.364–0.793)	0.17	24.11	0.33	39.27	(27.58–45. 47)
left bottom corner	0.67	0.26	0.61	0.19	0.39	(0.070–0.640)	0.15	23.54	0.29	30.14	(22.27–36.72)

total	0.66	0.23	0.60	0.22	0.65	(0.403–0.810)	0.12	19.43	0.24	32.89	(23.95–39.50)

advanced											
centered	1.93	0.52	1.80	0.50	0.84	(0.657–0.923)	0.20	10.54	0.39	26.35	(19.85–32.71)
right top corner	2.06	0.50	1.86	0.40	0.67	(0.327–0.842)	0.24	12.29	0.47	21.45	(26.49–27.19)
left bottom corner	1.95	0.44	1.84	0.37	0.69	(0.449–0.832)	0.21	11.15	0.41	19.89	(15.38–25.36)
total	1.98	0.46	1.83	0.40	0.84	(0.517–0.932)	0.17	8.81	0.33	21.68	(16.65–27.46)

**COPe [°] Dynamic Conditions**											

support											
horizontal moved	2.85	0.60	2.96	0.65	0.51	(0.220–0.713)	0.38	13.14	0.75	18.69	(15.15–24.41)
clockw. rotation	3.25	0.72	3.50	0.75	0.41	(0.112–0.645)	0.48	14.10	0.93	18.35	(14.87–23.97)
const. movement	1.54	0.48	1.29	0.28	0.18	(−0.098–0.457)	0.27	19.41	0.54	21.49	(16.40–26.43)

total	2.54	0.47	2.58	0.45	0.44	(0.135–0.672)	0.41	15.99	0.80	15.29	(12.39–19.97)

independent											
horizontal moved	4.02	0.92	3.43	0.80	0.51	(0.082–0.754)	0.54	14.59	1.06	20.90	(14.90–24.01)
clockw. rotation	4.09	1.01	3.75	1.01	0.48	(0.191–0.692)	0.63	16.13	1.24	22.32	(15.81–25.48)
const. movement	2.55	0.62	2.28	0.54	0.69	(0.327–0.852)	0.30	12.51	0.59	22.44	(16.73–26.96)

total	3.55	0.74	3.15	0.62	0.61	(0.176–0.816)	0.39	11.70	0.77	18.78	(13.80–22.24)

advanced											
horizontal moved	5.24	1.08	4.87	0.92	0.64	(0.374–0.805)	0.55	10.91	1.08	18.20	(13.82–22.27)
clockw. rotation	4.76	0.88	4.38	0.68	0.41	(0.109–0.650)	0.51	11.26	1.01	14.71	(11.41–18.39)
const. movement	4.03	0.74	3.56	0.49	0.54	(0.027–0.785)	0.39	10.34	0.77	15.17	(11.83–19. 07)

total	4.68	0.74	4.27	0.56	0.64	(0.124–0.841)	0.37	8.30	0.73	13.73	(10.64–17.15)

COPe: center of pressure error; ICC: intraclass correlation coefficient; CI: 95% confidence interval; SEM: standard error of measurement; MDC: minimal detectable change; CV: coefficient of variation; clockw.: clockwise; const.: constant. total: summary of the three different exercises. Spearman's rank correlation coefficient in absence of normal distribution:

** significance level (*p* < 0.01);

* significance level (*p* < 0.05)

**Table 4.** Analyses of the ABS in comparison with BBS in static and dynamic conditions (*n* = 29).

**ABS**				**BBS**				
		
**Parameter**		***M***	***SD***	**Parameter**	***M***	***SD***	***r***	***P***
		
static conditions								
TITC	[s]	28.35	1.53	OSI	2.23	0.78	**−0.52**^†^	0.004
COPe absolute	[°]	138,142	44,187	OSI	2.23	0.78	**0.60**	<0.001
COPe pitch x	[°]	75,935	26,751	APSI	1.79	0.64	**0.60**^†^	<0.001
COPe roll y [°]	[°]	62,207	23,778	MLSI	1.54	0.51	**0.62**	<0.001
dynamic conditions								
TITC	[s]	44.70	4.82	time [s]	95.00	28.14	−**0.72**^†^	<0.001
COPe absolute	[°]	2,599,972	174,034	time [s]	95.00	28.14	0.46 ^†^	0.012

ABS: Active Balance System; BBS: Biodex Balance System; COPe: center of pressure error, absolute, in roll and pitch directions; OSI: overall stability index; APSI: anterior-posterior stability index; MLSI: medial-lateral stability index; *r:* Pearson's rank correlation coefficient;

† Spearman's rank correlation coefficient; Significance level (*p* < 0.01) are in boldface.

## Supplementary Files

Supplementary File 1 ZIP-Document (ZIP, 1730 KB)
